# Amiodarone-induced Hyponatremia: A Case Report and a Review of the Literature

**DOI:** 10.19102/icrm.2018.090303

**Published:** 2018-03-15

**Authors:** Waseem Barham, Samer A. Zeayter, Abdulrahman Safadi, Ranjan K. Thakur

**Affiliations:** ^1^Department of Cardiology, Michigan State University, Lansing, MI, USA; ^2^Department of Nephrology, Michigan State University, Lansing, MI, USA

**Keywords:** Amiodarone, hyponatremia, secretion of antidiuretic hormone

## Abstract

Amiodarone is a widely used medication for controlling various types of cardiac arrhythmias. Nonetheless, it carries several known adverse effects that may preclude its use or necessitate discontinuation. Hyponatremia resulting from amiodarone is rarely reported, and its incidence is unknown. We present a case of severe hyponatremia secondary to amiodarone therapy and a review of the literature.

## Case presentation

A 79-year-old Caucasian female presented to the emergency department with persistent shortness of breath and rapid irregular palpitations that had been ongoing for seven hours. She had a past medical history of coronary artery disease, severe aortic stenosis, essential hypertension, and dyslipidemia, and she had undergone an angioplasty without stents 13 years prior. A physical examination revealed an initial blood pressure of 71/50 mmHg [heart rate: 132 beats per minute (bpm)] that spontaneously increased to 107/61 mmHg (heart rate: 111 bpm) within five minutes, an irregularly irregular pulse, oxygen saturation of 95% on room air, variable S1 and S2 sounds with a 3/6 systolic ejection murmur in the left upper sternal region, and fine end-inspiratory pulmonary crackles at the bilateral bases. No peripheral edema was noted. A twelve-lead electrocardiogram revealed atrial fibrillation and rapid ventricular response at an average rate of 144 bpm. A portable chest X-ray showed increased pulmonary vasculature markings along with air bronchograms and subtle bilateral costophrenic angle obliteration. The patient’s laboratory workup upon admission is shown in **Table 1**. Intravenous heparin was started for anticoagulation. Given the clinical picture of pulmonary edema, urgent electrical cardioversion was delivered, resulting in restoration of sinus rhythm. A subsequent two-dimensional echocardiogram showed preserved left ventricular function (estimated ejection fraction: 55%–60%), moderate left ventricular hypertrophy, a severely dilated left atrium, and severe aortic and moderate mitral valve stenosis. Amiodarone was started with oral loading at a dose of 400 mg three times per day, beginning on the first day of admission.

**Figure 1:**
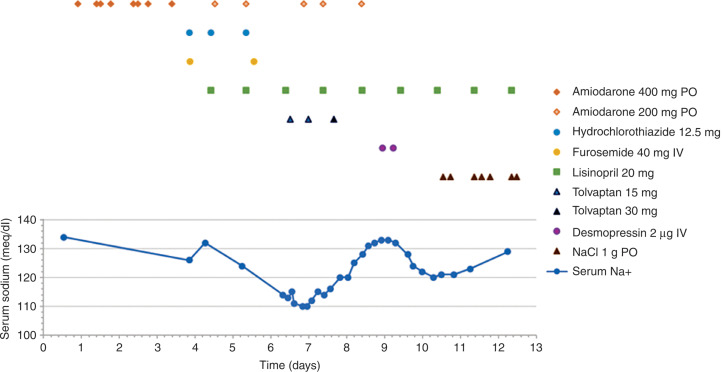
Serum sodium levels and related medication changes during hospitalization. The actual timing of each medication dose administration is marked by the corresponding legend symbol distributed over the x-axis. IV: intravenously; PO: per oral/orally.

On the fourth day following admission, the patient developed mild shortness of breath. A repeated chest X-ray showed a case of pulmonary congestion not significantly altered from the previous one. A repeated brain natriuretic peptide test showed the level was increased to 438 pg/ml. Her sodium (Na) was noted to have dropped from 134 meq/l to 126 meq/l. She was treated with a 40-mg dose of furosemide intravenously and her home regimen of hydrochlorothiazide (HCTZ), which had been discontinued upon admission, was resumed. This resulted in a subsequent improvement of her symptoms. The amiodarone was reduced to 400 mg daily; then, on hospitalization the fifth day, it was further reduced to 200 mg daily. On the sixth day, the patient began to complain of nausea, generalized weakness, headache, and blurred vision. A physical examination revealed a distended jugular vein, pulmonary rales, and the same systolic murmur noted previously. Peripheral edema remained absent. A computed tomogram of the head ruled out acute intracranial changes. Repeat serum Na level result was 124 meq/l, and HCTZ was discontinued. However, the patient’s Na level continued to drop throughout the next day (the seventh day) to 113 meq/l, reaching a nadir of 110 meq/l in less than 24 hours. Serum and urine osmolarities were 258 Osm/kg and 246 Osm/kg, respectively. Random urine Na, uric acid, and creatinine were tested **(Table 2)**. The fractional excretions of Na and urate (FEurate) were 0.36% and 19.6%, respectively. At that point, syndrome of inappropriate secretion of antidiuretic hormone (SIADH) was suspected. Other causes of hyponatremia, such as acute kidney injury, hypothyroidism, and adrenal insufficiency were ruled out **(Table 2)**. The patient’s intake of fluid was restricted and, due to the severe hyponatremia, she was treated with two 15-mg doses of tolvaptan spaced 12 hours apart. Due to a lack of improvement initially, an additional single dose of 30 mg was administered on the following day (the eighth day). An increase in serum Na level by 15 meq/l was subsequently noted in the following 24 hours and due to concern for potential inadvertent complications from rapid correction, dextrose 5% water was infused at 100 ml/h for 24 hours and two doses of desmopressin 2 µg were administered intravenously six hours apart. The rise in serum Na was successfully halted and instead it dropped to a level of 120 meq/l on day 10. As it has been linked to SIADH in very rare case reports, the amiodarone was suspected to have been the offending agent and was discontinued on the ninth day. Instead, sotalol was initiated 48 hours later for the maintenance of sinus rhythm. Sodium chloride (NaCl) tablets (3 g/day) were added and her serum Na level gradually improved within a few days to 129 meq/l on day 13 and 135 meq/l on day 18 after discharge, respectively. The serum Na levels and administration times of potentially Na level-altering medications are depicted in **Figure 1**.

## Discussion

Amiodarone is an antiarrhythmic drug composed of iodinated benzofuran derivative that exhibits activity in each of the four Vaughn-Williams antiarrhythmic classes. It has long been studied and is widely used in the acute and maintenance treatment of many different types of supraventricular and ventricular tachyarrhythmias. It has shown greater efficacy and a lower incidence of proarrhythmic effects than those of class I or class III antiarrhythmic drugs.^1–3^ Its various intravenous, oral, and intraosseous routes of administration have added to its widespread, flexible use. The side effects of amiodarone, however, are various and variable, precluding its use in some cases. The most common side effect from intravenous amiodarone is hypotension, while gastrointestinal (eg, nausea, vomiting, constipation, anorexia) and central nervous system (eg, malaise, fatigue, tremor, incoordination, dizziness, abnormal gait, paresthesia) side effects are those most commonly reported with the use of its oral form. Thyroid dysfunction, pulmonary toxicity, optic neuropathy, and neuritis are other known potential side effects. Serious and sometimes fatal reactions from amiodarone use have also been reported, making it the least favorable antiarrhythmic drug, especially when other suitable options exist. Electrolyte disturbances (eg, hypokalemia and hypomagnesemia) may increase amiodarone’s arrhythmogenic effects; correction of such disturbances is advised prior to initiating therapy.^1^

Hyponatremia is an extremely rare side effect of amiodarone use, a fact which is often overlooked or under-recognized, especially in hospitalized patients who may have more than one plausible etiology for the condition. In our patient, the presence of other confounding etiologies, such as congestive heart failure and the use of diuretics, made her clinical picture murky and nearly obscured the effect of the amiodarone. However, careful evaluation of the temporal sequence of events made it clear that the hyponatremia was in fact induced by the amiodarone. The patient’s Na level had been previously stable for months, but dropped following the introduction of the amiodarone and prior to any use of diuretics. Both thiazide and loop diuretics were administered right after the hyponatremia was first detected. Thiazides are distal tubule diuretics that can cause hyponatremia through promoting Na excretion and water reabsorption independent of the antidiuretic hormone (ADH).^4^ However, hyponatremia with loop diuretic use is unusual due to the reduced responsiveness to ADH that accompanies increased ADH levels induced by volume depletion.^5^ Although both thiazide and loop diuretics can be associated with hyponatremia, the lower level of blood urea nitrogen (BUN) present in comparison with that at admission, as well as the hypouricemia (normal: < 4 mg/dl) noted on the fourth day, were not in favor of this assumption. In addition, the abnormally high uric acid clearance quantified by the elevated FEurate level (normal: 4%–11%) was highly suggestive of SIADH.^6^ In contrast with Na and urea, uric acid is exclusively handled in the proximal tubule and a direct interaction with common diuretics is not expected. Elevated FEurate is more superior to hyponatremia and hypouricemia alone in the diagnosis of SIADH, even in patients in whom diuretics have been used.^7^ After excluding the possibilities of acute kidney injury, hypothyroidism, and adrenal insufficiency, amiodarone was suspected to have caused the SIADH picture in the current case.

In the published medical literature, only 17 cases of amiodarone-induced hyponatremia have been reported, to our knowledge **(Table 3)**.^8–21^ The affected patients were predominantly male (n = 13) and elderly (range: 58–88 years, median: 68 years). Either atrial fibrillation or ventricular arrhythmia was the indication to start amiodarone in all 17 cases. Additionally, most of the patients had multiple comorbidities, and none of them had lone atrial fibrillation. Interestingly, the onset of hyponatremia varied from occurring at one day to six months following the initiation of amiodarone therapy, which was performed using either an intravenous or oral formulation. The level of hyponatremia at presentation ranged from 107 meq/l to 131 meq/l, with the lowest reported nadir being 105 meq/dl. Symptoms, which invariably were present at levels less than 130 meq/dl, were non-specific and frequently included altered mental status, generalized physical weakness, nausea, and abdominal pain. The type of symptoms that presented, however, did not seem to have any correlation with the severity of hyponatremia. In all cases, SIADH was suggested as the most likely etiology, and the diagnosis was made based on serum and urine osmolarity and the identification of Na levels consistent with SIADH after ruling out other differential diagnoses, such as renal failure, adrenal insufficiency, and decompensated thyroid function. Amiodarone was eventually stopped in 14 patients, while the dose was decreased in the other three. Other steps were undertaken in managing hyponatremia in some of the cases, such as fluid restriction, hypertonic saline infusion, and demeclocycline administration. Tolvaptan, a non-peptide vasopressin receptor antagonist, was tried in one patient without any noted improvement indicated by serum Na level.^18,22^ Another patient received hemodialysis.^16^ Regardless of the management approach, all of the patients eventually experienced normalization of their serum Na levels within nine days to one month. One of the patients who was managed only with dose reduction developed recurrence of hyponatremia after reloading with amiodarone.^12^ In that case, the hyponatremia improved and eventually resolved again after resuming the previous dose of amiodarone.

In our case, the patient was a frail, elderly female who developed moderate hyponatremia within three days and critical symptomatic hyponatremia within six days of oral amiodarone use, respectively. When the hyponatremia was first found on the third day, a total of 3,200 mg of amiodarone had been administered. An additional 600 mg (total: 3,800 mg) of amiodarone was administered before the hyponatremia reached its nadir of 110 meq/l on the sixth day and symptoms started to develop. This necessitated the undertaking of other actions before amiodarone was suspected as the offending agent and discontinued. Tolvaptan (total: 60 mg) was given, with an onset time of action of approximately 13 hours. The recurrence of hyponatremia on day 10 was likely due to the preceding use of desmopressin and dextrose 5% water (to lessen the rapid correction of serum Na). Although normalization (135 meq/l) of the serum Na was achieved nine days after discontinuation, the exact time needed to reverse the hyponatremic effect of the amiodarone is not clearly known in this case due to the implementation of other treatment actions. Whether the withdrawal of amiodarone alone could have led to hyponatremia resolution or not is unclear.

The mechanism by which amiodarone causes hyponatremia remains unknown. Hypothetical explanations have been suggested, such as a direct effect on the kidneys, ADH secretion and/or the presence of its receptors in the kidneys, and thyroid function. Iovino et al. suggested amiodarone, through its action on ion channels—mainly calcium channels—might stimulate insertion of an aquaporin-2 water channel into the apical membrane of the renal collecting duct, resulting in dilutional hyponatremia.^19^ However, the rarity of this side effect may suggest a prerequisite genetic predisposition or polymorphism. Further research is yet to be done to investigate the potential mechanisms and drug targets leading to hyponatremia.

Shedding light on this rare, likely overlooked, side effect of amiodarone therapy is important for several reasons. The level of hyponatremia is often moderate to severe, and frequently symptomatic, in nature. Moreover, patients receiving this drug are usually elderly and vulnerable and have other medical reasons that may account for hyponatremia. Although amiodarone has a lengthy side effect profile, it is still widely used and reserved for cases in which other antiarrhythmic medications are considered less effective or contraindicated. While stopping amiodarone may help to reverse the hyponatremia, finding an alternative antiarrhythmic medication to use may be challenging. Lowering the dose of the amiodarone could be an alternative option, rather than discontinuing it entirely; however, further research is still needed.

## Conclusions

Amiodarone is a rare cause of hyponatremia that can acutely manifest and rapidly reach critical levels. Although the exact mechanism by which amiodarone can induce hyponatremia remains unclear, SIADH appears to be a common denominator among all the reported cases, including ours. Further research is needed to explore the incidence and impact of this adverse effect of amiodarone. Furthermore, the best strategy to manage hyponatremia due to amiodarone therapy is still undefined, and challenges can arise if other comorbidities are present. The most important step, however, remains to have a low index of suspicion for suspecting amiodarone as the culprit.

## Figures and Tables

**Table 1:** Selected Laboratory Test Results on Hospital Presentation

Test (Serum)	Value	Local Reference Range
Sodium	134 meq/l	135–145 meq/l
Potassium	4.4 meq/l	3.5–4.9 meq/l
Chloride	101 meq/l	96–110 meq/l
Bicarbonate	29.0 mmol/l	20.0–32.0 mmol/l
Glucose	116 mg/dl	65–99 mg/dl
Urea	27 mg/dl	6–23 mg/dl
Creatinine	1.09 mg/dl	0.60–1.40 mg/dl
GFR estimation	48 ml/min	> 90 mg/dl*
Total bilirubin	0.3 mg/dl	0.2–1.2 mg/dl
Albumin	3.9 g/dl	3.6–5.0 g/dl
Aspartate aminotransferase	31 U/l	10–40 U/l
Alanine aminotransferase	18 U/l	3–45 U/l
Alkaline phosphatase	41 U/l	0–120 U/l
Prothrombin time, INR	9.9 s, 1.0	9.0–11.5 s
Troponin	0.08 ng/ml	0.00–0.03 ng/ml
Brain natriuretic peptide	215 pg/ml	0–100 pg/ml
Thyroid-stimulating hormone	1.29 IU/ml	0.35–4.01 IU/ml

GFR: glomerular filtration rate; INR: international normalized ratio.

*Non-African-American.

**Table 2:** Results of Laboratory Tests for Hyponatremia on the Seventh Day of Hospitalization

Test	Value	Local Reference Range
Serum sodium	113 meq/l	135–145 meq/l
Serum potassium	3.9 meq/l	3.5–4.9 meq/l
Serum chloride	78 meq/l	96–110 meq/l
Serum bicarbonate	29.0 mmol/l	20.0–32.0 mmol/l
Blood urea nitrogen	10 mg/dl	6–23 mg/dl
Serum creatinine	0.74 mg/dl	0.60–1.40 mg/dl
Estimated GFR	76 ml/min	> 90 mg/dl*
Serum uric acid	2.9 mg/dl	2.5–7.0 mg/dl
Serum morning cortisol	36.3 µg/dl	4.3–19.8 µg/dl
Thyroid-stimulating hormone	2.62 IU/ml	0.35–4.01 IU/ml
Serum osmolality	258 Osm/l	280–295 Osm/l
Urine osmolality	246 Osm/kg	250–1,200 Osm/kg
Urine specific gravity	1.005	1.003–1.030
Random urine creatinine	87.3 mg/ml	N/A
Random urine sodium	49 meq/ml	N/A
Random urine uric acid	67.1 mg/dl	N/A
Fractional excretion of sodium	0.36%	N/A
Fractional excretion of uric acid	19.6%	N/A

GFR: glomerular filtration rate; N/A: not available.

*Non-African-American.

**Table 3:** Summary of Amiodarone-induced Hyponatremia Peer-reviewed Published Case Reports

Study	Age, Sex	Amiodarone Indication	Amiodarone Dose and Route	Baseline Na	Hyponatremia Symptoms	Onset of Hyponatremia Postamiodarone, Na (meq/l)	Nadir of Hyponatremia Postamiodarone, Na (meq/l)	Mechanism	Treatment	Day of First Na Increase and Trend, Na (meq/l)
Muñoz Ruiz et al.^8^	67 years, female	AF	N/A	N/A	Yes	4 months (110 meq/l)	N/A	SIADH	Amiodarone discontinued	N/A
Odeh et al.^9^	62 years, female	AF	300 mg PO daily	N/A	Yes	6 months (120 meq/l)	N/A	SIADH	Fluid restriction, amiodarone discontinued	Day 5 (133 meq/l) Day 14 (143 meq/l)
Ikegami et al.^10^ (case 1)	63 years, male	VA	800 mg PO daily	135 meq/l	Yes	7 days (119 meq/l)	7 days (119 meq/l)	SIADH	Dose decrease to 100 mg daily	Day 22 (122 meq/l)
Ikegami et al.^10^ (case 2)	83 years, male	VA	1,400 mg PO daily	140 meq/l	Yes	7 days (121 meq/l)	7 days (121 meq/l)	SIADH	Fluid restriction, dose decrease to 100 mg PO daily	Day 14 (133 meq/l)
Patel and Kasiar^11^	67 years, male	VA	200 mg PO daily	134 meq/l	Yes	3 weeks (126 meq/l)	3 months (117 meq/l)	SIADH	Fluid restriction, amiodarone discontinued	Day 3 (129 meq/l) Day 30 (136 meq/l)
Aslam et al.^12^	72 years, male	VA	2 g PO loading	136 meq/l	NA	2 days (N/A)	4 days (117 meq/l)	SIADH	Fluid restriction, dose decrease to 200 mg PO daily	Day 2 (120 meq/l) Day 14 (130–135 meq/l)
Shavit and Sherer^13^	85 years, male	AF	N/A	N/A	Yes	30 days (122 meq/l)	N/A	SIADH	Amiodarone discontinued	“Normal in a few days”
Paydas et al.^14^	58 years, male	N/A	N/A, PO	NL	Yes	5 months (107 meq/l)	N/A	SIADH	Amiodarone discontinued	Day 14 (130 meq/l)
Afshinnia et al.^15^	66 years, male	VA	150 mg IV bolus + drip	138 meq/l	No	2 days (131 meq/l)	7 days (116 meq/l)	SIADH	Amiodarone discontinued	Day 2 (120 meq/l) Day 9 (133 meq/l)
Singla et al.^16^	58 years, male	VA	IV loading then 1,200 PO daily	132 meq/l	No	3 days (120 meq/l)	4 days (118 meq/l)	SIADH	Amiodarone discontinued, Hemodialysis	N/A
Pham et al.^17^	84 years, male	AF	IV and PO loading then 400 mg daily	140 meq/l	Yes	11 days (120 meq/l)	17 days (105 meq/l)	SIADH	Fluid restriction, amiodarone discontinued, 3% saline, demeclocycline	Day 1 (110 meq/l) Day 10 (130 meq/l)
Dutta et al.^18^	63 years, male	VA	100 mg PO daily	N/A	Yes	2 months (124 meq/l)	9 months (109 meq/l)	SIADH	Fluid restriction, NaCl PO, tolvaptan, amiodarone discontinued	Day 3 (122 meq/l)
Iovino et al.^19^ (case 1)	68 years, male	AF	1,050 mg IV then 200 mg PO daily	134 meq/l	Yes	10 days (118 meq/l)	10 days (118 meq/l)	SIADH	Fluid restriction, amiodarone discontinued, 3% saline	Day 1 (120 meq/l) Day 3 (133 meq/l)
Iovino et al.^19^ (case 2)	72 years, male	AF	1,050 mg IV then 200 mg PO daily	136 meq/l	Yes	12 days (120 meq/l)	12 days (120 meq/l)	SIADH	Fluid restriction, amiodarone discontinued, 3% saline	Day 1 (125 meq/l) Day 3 (138 meq/l)
Maloberti et al.^20^	78 years, male	VA	200 mg PO daily	NL	Yes	5 years (128 meq/l)	5 years (110 meq/l)	SIADH	Fluid restriction, amiodarone discontinued, 3% saline	Day 1 (120 meq/l) Day 5 (134 meq/l)
Oner et al.^21^	88 years, female	AF	1,050 mg IV then 200 mg PO daily	N/A	Yes	3 days (111 meq/l)	N/A	SIADH	Fluid restriction, amiodarone discontinued, 3% saline	Day 1 (113 meq/l) Day 5 (130 meq/l)
Barham et al. (current case)	79 years, female	AF	1,200 mg PO daily	134 meq/l	Yes	3 days (126 meq/l)	7 days (110 meq/l)	SIADH	Fluid restriction, amiodarone discontinued, tolvaptan, NaCl PO	Day 7 (112 meq/l) Day 18 (135 meq/l)

Na: sodium; AF: atrial fibrillation; VA: ventricular arrhythmia; PO: per oral/orally; IV: intravenously; SIADH: secretion of antidiuretic hormone; NaCl: sodium chloride.
